# Medical Society of the County of Erie

**Published:** 1906-08

**Authors:** Franklin C. Gram


### SOCIETY PROCEEDINGS.

### Medical Society of the County of Erie

### Semiannual Meeting, June 12, 1906.

Reported By FRANKLIN C. GRAM, M. D., Secretary.

The semiannual meeting of the Medical Society of the County of Erie was held in the rooms of the Society of Natural Sciences, Buffalo Library Building, Tuesday, June 12, 1906, being called to order at 10.30 A. M. by the president, Dr. A. H. Briggs.

On motion of Dr. Charles A. Wall the reading of the minutes of the annual meeting, and of the special meeting, held April 3,

1906, to consider pending medical legislation, was dispensed with and they were approved. The minutes of the special meeting held April 18, 1906, to consider the new by-laws, were read and approved, on motion of Dr. Hopkins. The circular appeal by the Journal of the American Medical Association on behalf of the San Francisco flood sufferers, was received and filed.

The secretary read the resignations of M. A. Crockett and Julius Pohlman as members of this society. The resignation of Dr. Crockett was accepted, as he has removed from the State, but, on motion of Dr. Hopkins, that of Dr Pohlman was received and laid on the table. The treasurer read a communication from H. H. Bingham, stating that he is not a member of this society. This communication was likewise ordered tabled. The resignation of Dr. Leonard was accepted, as the doctor now resides in Porto Rico.

The treasurer read communications from Dr. A. J. Colton and Dr. George N. Jack, in which they stated that they were members of the Erie County Medical Association and had paid their dues to date in said association. They desired to be released from paying back dues in the Medical Society of the County of Erie, to which they also belonged. Dr. Hopkins moved that the treasurer remit the back dues of Drs. Colton and Jack.

Dr. F. F. Hoyer objected; he stated that he likewise belonged to the society and the association and had regularly paid his dues in each. There were many other physicians who also belonged to both organizations, and it would be unfair to them to remit the dues of those who had neglected to pay in one or the other. Dr. Hopkins said it was not a question of right, but of expediency, and he had made his motion to avoid friction. Dr. Hoyer then withdrew his objections and the motion was adopted.

The treasurer announced that he had a number of volumes of the state society transactions for 1904-1905. On motion he was directed to distribute them among the members.

Dr. Wall, being the only member present from the Committee on Membership, requested the chair to appoint temporary substitutes. The president appointed Drs. F. F. Hoyer and J. H. Grant. This committee reported favorably upon the applications of Drs. William M. Mehl, 71 E. Genesee St.; Dr. Julius Richter, 1345 Clinton St.; H. Arnold Pierce, Blasdell, N. Y.; and Herman K. DeGroat, 267 Carolina St. On motion, the secretary was directed to cast the ballot of the society in favor of these applicants, and they were declared duly elected.

The treasurer desired to know how much to collect from these new members. On motion of Drs. Gram and Krauss he was directed to collect one dollar county dues and three dollars state dues, for 1906. The new members were then introduced to the society by President Briggs.

Dr. William C. Callanan, who had been appointed for that purpose, read the following memorial of Dr. John J. Walsh:

IN MEMORIAM—JOHN J. WALSH.

Dr. John J. Walsh was the son of Dr. Nicholas Walsh and was born in Ireland, December 8, 1848. The following year his parents came to the United States. His father was at one time Coroner of Erie County. When a young man, Dr. Walsh first took up the study of law, but later turned to medicine and for a time he was a sanitary inspector in the department of health. He has served as medical examiner in many of the German branches of the C. M. B. A., was secretary of the alumni association of the Medical Department of the University of Buffalo and was connected with the medical staff of the Sisters' of Charity Hospital.

Dr. Walsh was amember of the Medical Society of the State of New York, the Medical Society of the County of Erie, of the C. M. B. A., of the Knights of Columbus, of the Buffalo Catholic Institute and several other organizations. He is survived by a son, Attorney Walter B. Walsh, two brothers, Edward F. and Louis C. Walsh, and one sister, Mary J. Walsh. He died December 6, 1905.

The memorial was adopted and ordered to be spread upon the minutes.

Dr. William C. Krauss stated that Dr. F. F. Hoyer had been a member of this society for many years and moved that he be made an honorary member. The motion was unanimously adopted. Dr. Hoyer thanked the society for this great compliment, saying that he had practised medicine for about 60 years and had been a member of this society for 50 years.

Dr. Wall said that provision for honorary membership should be made in the new by-laws. He was directed to draft such a section. Dr. Wall then presented the new by-laws, as adopted at the meeting of April 16, 1906.

The secretary read a letter from the Medical Society of the State of New York, signed by the President and Secretary, approving the proposed by-laws. Dr. Wall moved the adoption of the by-laws, with the exception of several corrections which he noted.

Dr. Hopkins objected to the proceedings and stated his reasons. The society may adopt any by-laws, but these must be in accordance to the statutes and laws of the State of New York. He asked the society not to adopt by-laws which are at variance with the laws of the State.

Dr. Wall said that the legislature has the power to undo acts of previous legislatures and create an entirely new act. This was done by the legislature. He read the act which amalgamated the society and the association.

Dr. Hopkins said this statute simply authorized the amalgamation of the society and the association, but did not authorize any change in the medical law. There is no reason why the laws which were in force for 85 years are not good now.

Dr. Cohen raised the point of order that the legal question involved had been settled by competent authorities. Dr. Hopkins asked to have the act read, which authorized the amalgamation. Dr. Wall then read the act. Dr. Hopkins insisted that this simply reenacted existing laws and offered the following resolution, which was seconded by Dr. Blaauw, and adopted:

Resolved, That the by-laws reported by the committee, as printed and corrected, be submitted to the counsel of this society for examination, and opinion as to whether they conflict in any manner with the statutes of the State of New York now in force, and that such report of counsel be made at an adjourned meeting on the second Monday in October, at an evening session, the place to be subject to the call of the Chair.

At 12:10 the society adjourned until the second Monday evening in October.

